# Completed Suicide With Violent and Non-violent Methods by the Elderly in Rural China: A Psychological Autopsy Study

**DOI:** 10.3389/fpsyt.2021.624398

**Published:** 2021-06-15

**Authors:** Rong-Ting Zhu, Zhen-Yu Ma, Cun-Xian Jia, Liang Zhou

**Affiliations:** ^1^Guangzhou Brain Hospital, Guangzhou Medical University, Guangzhou, China; ^2^School of Public Health, Guangxi Medical University, Nanning, China; ^3^Department of Epidemiology, School of Public Health, Shandong University, Jinan, China

**Keywords:** suicide, violent method, non-violent method, rural, elderly

## Abstract

**Background:** Late-life suicide is a severe public health problem in rural China; however, knowledge regarding the specific characteristics and risk factors for completed suicide via violent and non-violent methods among elderly individuals in rural China is limited.

**Methods:** Subjects aged 60 years or older were selected from rural areas in Shandong, Hunan, and Guangxi provinces in China. This study was a 1:1 matched case-control design conducted by using the psychological autopsy method.

**Results:** According to the univariate analyses, the presence of mental disorder, higher degree of depression, loneliness, lack of social support, hopelessness, impulsivity, and increased quantity of life events were associated with suicide in both violent and non-violent methods. For violent suicide, another risk factor was the lack of pesticides at home. For non-violent suicide, other risk factors were not currently married, family suicide history, and alcohol use disorder (*P* < 0.05). Variables that remained in the logistic regression model were the severity of depressive symptoms for both violent and non-violent suicide. For non-violent suicide, the degree of hopelessness was another independent risk factor. In addition, violent suicides were more likely to lack pesticides at home, choose the spring season and have an increased quantity of life events than those who died by suicide via non-violent methods.

**Conclusion:** The major risk factor for both violent and non-violent suicide was the severity of depressive symptoms. Suicide prevention measures that focus on depression among this vulnerable population are urgently needed. Moreover, the characteristics of suicides via violent methods differed from those via non-violent methods among elderly individuals. Suicide prevention efforts should be tailored to the specific characteristics of the different suicide methods utilized by older adults in rural areas.

## Introduction

Suicide represents a severe public health problem in China and worldwide. According to data from the World Health Organization (WHO), the burden of suicide has shifted from Western to Eastern Europe over the past 50 years and currently appears to be the highest in Asia (1). The suicide rates among elderly individuals have been reported in several studies (2); older men and women (aged 65 or older) exhibit the highest suicide rates in almost all countries, reaching 34.5/100,000 in China (6.5 times more than the rate in the population younger than 65 years), and elderly people who live in rural areas are more likely to die by suicide than those who live in urban areas (incidence rate ratio = 1.83) (3).

Late-life suicide is a multidimensional behavior that has special characteristics and risk factors. Research has found that unemployment, not married, mental disorders, and depressive symptoms were independent risk factors related to suicide in older adults in rural China (4). Feeling left behind can elevate suicide risk due to increasing life stresses, mental disorders, and depressive symptoms and decreasing social support (4). Studies have shown that older adults with high suicide intent tend to choose highly lethal suicide methods and are well-prepared (5, 6). Investigating the specific characteristics of suicide methods among older adults is critical for suicide prevention.

Studies have found the remarkable differences of certain patterns of suicide methods in different countries. Jumping from high locations is a frequently used suicide method among older adults in New York (7), while more than half of elderly individuals who died by suicide used firearms in the USA (8). Hanging is the predominant method among older adults in Turkey (9). The choice of suicide method among elderly individuals is not random and depends on multiple factors, not only culture social-cultural acceptability, but also availability of suicide means. The ingestion of pesticides is the most common method among elderly individuals in rural China because of the high accessibility to pesticides (10). Furthermore, it was found that the method used at an unsuccessful suicide attempt may predict later completed suicide, suggesting study on suicide methods is of much significance in suicide prevention (11). A popular dichotomy of suicide methods is the characterization of methods as violent or non-violent (12): hanging, jumping from high locations, use of a firearm or shotgun, cutting and piercing with sharp objects, and getting run over by a train or other vehicle are classified as violent methods, whereas the ingestion of pesticides, overdose of drugs, poisoning by gases, and suffocation are classified as non-violent methods. Some sociodemographic and clinical characteristics are related to the choice of suicide method (13). A study of European populations found that males were more likely to choose violent and highly lethal methods than females (14), which is similar to the findings of studies on the Japanese population (15). Nevertheless, knowledge regarding the characteristics of violent and non-violent suicide methods in elderly individuals in rural China is limited.

In this study, we focused on investigating the differences between violent suicide methods and non-violent suicide methods among elderly individuals in rural China. Psychological autopsy was performed to compare the characteristics of individuals who utilized these two types of suicide methods, and the risk factors potentially related to violent suicide methods were examined.

## Methods

### Sample and Sampling

A multistage stratified cluster sampling method was used to select research sites. All 31 provinces in mainland China were classified into three strata based on the GDP per capita; Shandong, Hunan, and Guangxi were selected from each stratum. The counties in the three provinces were also stratified into three strata based on average income. Twelve counties were randomly selected from the three provinces (one county in each stratum in Shandong and Hunan provinces and two counties in each stratum in Guangxi Province).

The study was a 1:1 matched case-control design. A consecutive sampling method was used to obtain the data, and 242 suicide cases aged 60 and above who died from suicide were entered into the study. One living control person was randomly chosen from the same or neighboring rural village for each suicide case, with the same gender and birth year (± 3 years). All village doctors and local public health professionals involved in the death certification process were briefly trained and were required to report all suicides in older adults to the local Center for Disease Control and Prevention.

In this study, we divided all the suicide methods into a violent group (violent suicide cases and violent living controls included) and a non-violent group (non-violent suicide cases and non-violent living controls included). The violent suicide methods included hanging, drowning, jumping, and wrist cutting, whereas the non-violent suicide methods included the ingestion of pesticides or other poisons (16, 17).

### Study Procedures

This study was conducted from June 2014 to September 2015. All the interviewers were intensively trained on the determination of manner of death, method of psychological autopsy, interview techniques, and instruments used.

When a suicide case was reported, interviews with informants of the suicide case were arranged 2–6 months later. The proxy data for suicide victims were provided by two informants: generally, the first informant was the next-of-kin who lived with the suicide victim, and the second informant was always a friend, a neighbor, or a remote relative. Each informant was interviewed separately by one trained interviewer. The average interview time was 90 min. In the interview process, to obtain the most accurate information on the target person and prevent the family members of the suicide victims from concealing the real situation, the second informant was interviewed at the same time as the first informant; the two informants were consistent in the survey time but were separated by survey location to avoid mutual interference. After the interview, the questionnaire was examined and completed by the interviewer, and then the questionnaire was submitted to the quality control person. The details of the interview procedures can be found in a previous study (4).

This study was approved by the Institutional Review Boards of Shandong University, Central South University, and Guangxi Medical University. Written informed consent was obtained from all who provided information about the individuals who died by suicide. Participants in the living control group and all the informants of living comparisons and suicide cases provided written informed consent to participate in this study.

### Instruments and Recorded Measurements

#### Demographic Characteristics

The sociodemographic factors included age, gender, education level, marital status (currently married status: married and lived together, unmarried cohabitation, or remarried; unstable marital status: single, divorced, widowed, or married but separated), annual family income, and the storage of pesticides at home.

#### Suicidal Behavior

The characteristics of suicidal behavior included previous suicide attempts and the timing, and season of the suicide.

#### Suicide Intent Scale (SIS)

The SIS is composed of an objective section (items 1–8) and a subjective portion (items 9–15) and is used to evaluate the degree of suicidal intent. In this study, we used the first eight items of the SIS to estimate the level of suicidal intent. The possible score ranges from 0 to 16, and a high score indicates a strong suicidal intent. The Chinese version of the SIS has been shown to have satisfactory reliability and validity (18).

#### Life Events Scale for the Elderly (LESE)

Stressful life events over the final 12 months before death or investigation were measured by the LESE, which covers 46 life events and was developed specifically for older adults in China (19). Each event is assessed based on the following 5 aspects: occurrence time, event property (positive/negative life events), degree of psychological impact, duration, and frequency of occurrence. In this study, quantity of life events (the degree of psychological impact of life events × duration × frequency of occurrence) was used to reflect the impact of life events on an individual. High scores indicate an increased impact due to life events.

University of California Los Angeles Loneliness Scale-6 (ULS-6) Loneliness was measured by the Chinese version of the ULS-6, which is a 6-item instrument translated by Zhou et al. (20). Each item is rated on a 4-point scale ranging from 1 to 4. The total score ranges from 6 to 24, and high scores represent high levels of loneliness. In a previous psychological autopsy study, the Chinese version of the ULS-6 showed good reliability and validity among elderly individuals in rural China (21).

#### Beck Hopelessness Scale (BHS-4)

The Chinese version of the BHS-4 is an excellent instrument used to measure the degree of hopelessness. It is composed of 4 items relevant to success, a dark future, breaks (out of luck), and faith. Each item is rated on a 5-point scale. The total score ranges from 4 to 20, and a high score represents a great degree of hopelessness. The BHS-4 has been shown to perform as well as the original 20-item scale (BHS-20) (22–24).

#### Barratt Impulsiveness Scale (BIS-11)

The BIS-11 is a self-assessment tool used to measure impulsiveness. The current version includes 30 items, which cover the following three main dimensions of impulsive behavior: attentional impulsiveness, motor impulsiveness, and non-planned impulsiveness. Each item response ranges from 1 (never) to 4 (almost always), with a high score indicating high impulsivity. The BIS-11 has sound reliability and validity and can be used in the social and cultural context of China (25).

#### Duke Social Support Index (DSSI)

The 23-item DSSI is used to measure the level of social support. The total score ranges from 11 to 45, and a high score indicates a high level of social support. The DSSI has shown good reliability and validity in previous psychological autopsy studies in China (26).

#### Geriatric Depression Scale (GDS)

The severity of depressive symptoms was assessed by the GDS, which consists of 30 items. The possible total score ranges from 0 to 30, and a high score indicates severe depressive symptoms. The severity of depressive symptoms is categorized into the following three grades: no or mild depression (0–10), moderate depression (11–20), and severe depression (>20). The GDS has been validated among elderly individuals in rural China (27, 28).

#### Mental Disorders

The Chinese version of the Structured Clinical Interview for the Diagnostic and Statistical Manual of Mental Disorders, Fourth Edition (DSM-IV; SCID) (29, 30), was used in this study to generate current diagnoses of mental disorders. Diagnoses were made by psychiatrists in consensus meetings during which all information from both informants and previous medical records was presented. The following four categories of diagnoses were included: mood disorders, schizophrenia and other psychotic disorders, alcohol dependence, and anxiety disorders. Diagnoses of personality disorders or rare disorders (e.g., mental disorder with onset in childhood or adolescence, illicit drug abuse, eating disorders) were not included. Multiple diagnoses were made if appropriate.

#### Statistical Analysis

All statistical analyses were conducted with the Statistical Package for the Social Sciences (SPSS for Windows, version 20.0, SPSS Inc., Chicago, IL, USA). Kolmogorov-Smirnov *Z*-tests or *T*-tests were used to compare continuous variables such as age, quantity of life events, and the scores from the SIS, BHS, ULS, BIS, DSSI, and GDS. Chi-square tests were used to compare categorical variables such as sociodemographic factors (including gender, marital status, annual family income, education level), pesticides stored at home, family suicide history, mental disorders, previous suicide attempts, suicide timing, and suicide season. Binary logistic regression models were used to analyze factors related to the use of violent and non-violent suicide methods. Both backward stepwise and enter method inclusion of variables in the conditional logistic regression equation were tested to identify the most stable model. The level of significance was *P* < 0.05 in all the statistical analyses.

## Results

In this study, the most common method of suicide was pesticide ingestion, which accounted for 125 cases (51.7%). The second most common method was hanging (95, 39.3%), followed by drowning (9, 3.7%), ingesting poisons other than pesticides (8, 3.3%), jumping from high locations (3, 1.2%) and wrist cutting (2, 0.8%).

### Demographic Characteristics

The demographic characteristics of suicide cases and living controls are summarized in Table 1. Those who died by suicide via violent methods were approximately 2 years older than those who died via non-violent methods (*P* = 0.033). No significant differences were observed in the sex distribution, marital status, education level, annual family income, and health conditions between the violent and non-violent suicide cases (all *P* > 0.05).

**Table 1:** Comparison of sociodemographic, psychological, social environment, and life event characteristics of suicide cases and living controls.

	**Violent**			**Non-violent**			**VCA vs. NCA**
**Variables**	**Suicide cases (*n* = 109)**	**Living controls (*n* = 109)**	**χ2/*t/z***	**Suicide cases (*n* = 133)**	**Living controls (*n* = 133)**	**χ2/*t/z***	**χ2/*t/z***
Men, *n* (%)	62 (56.9)	62 (56.9)		73 (54.9)	73 (54.9)		0.10
Age, mean (SD), yrs	75.7 (8.16)	75.0 (8.23)	0.653	73.4 (8.17)	73.3 (8.05)	0.098	−2.15^*^
Marital status, *n* (%)			3.764			18.13^***^	1.10
Currently married	59 (54.1)	73 (67.0)		63 (47.4)	97(72.9)		
Non-currently married	50 (45.9)	36 (33.0)		70 (52.6)	36 (27.1)		
Annual family income, *n* (%)			1.18			2.19	2.59
0–3599	37 (33.9)	36 (33.0)		49 (36.8)	38 (28.6)		
3600–9999	38 (34.9)	32 (29.4)		34 (25.6)	36 (27.1)		
>10001	34 (31.2)	41 (37.6)		50 (37.6)	59 (44.4)		
Education, *n* (%)			0.906			2.35	3.44
Below primary school	46 (42.2)	41 (37.6)		65 (48.9)	55 (41.4)		
Primary school	47 (43.1)	54 (49.5)		58 (43.6)	62 (46.6)		
Above primary school	16 (14.7)	14 (12.8)		10 (7.5)	16 (12.0)		
Pesticides stored at home, *n* (%)	42 (38.5)	57 (52.3)	4.163^*^	85 (63.9)	75 (56.4)	1.57	15.47^***^
Family suicide history, *n* (%)	29 (26.6)	22 (20.2)	1.254	33 (24.8)	15 (11.3)	8.24^**^	0.10
Life events quantity, median (QL,QU)	54 (31, 76)	35 (12, 64)	−6.77^***^	40 (21, 64)	16 (4, 33.5)	−7.22^***^	−2.38^*^
BIS total score, mean (SD)	97.66 (16.80)	86.67 (16.46)	4.88^***^	99.71 (16.51)	87.11 (14.11)	6.70^***^	0.95
DSSI total score, mean (SD)	22.63 (5.47)	27.46 (6.82)	−5.76^***^	23.09 (6.39)	27.48 (6.84)	−5.41^***^	0.59
GDS total score, mean (SD)	21.92 (6.27)	9.35 (6.54)	14.49^***^	20.98 (5.66)	9.12 (6.35)	16.10^***^	−1.22
BHS total score, mean (SD)	14.39 (2.61)	9.75 (2.96)	12.26^***^	14.68 (2.49)	9.58 (2.52)	16.62^***^	0.91
ULS total score, mean (SD)	15.33 (5.17)	11.06 (4.19)	6.71^***^	15.72 (4.88)	10.94 (3.87)	8.86^***^	0.61
Any DSM-IV Axis I diagnosis, *n* (%)	49 (45.0)	5 (4.6)	47.66^***^	73 (54.9)	7 (5.3)	77.87^***^	2.36
Mood disorders, *n* (%)	40 (36.7)	3 (2.8)	39.66^***^	62 (46.6)	5 (3.8)	64.82^***^	2.42
Psychotic disorders, *n* (%)	9 (8.3)	1 (0.9)	6.71^*^	6 (4.5)	0 (0)	6.14^*^	1.45
Alcohol use disorder, *n* (%)	4 (3.7)	1 (0.9)	1.84	10 (7.5)	1 (0.8)	7.68^**^	1.63
Anxiety disorders, *n* (%)	4 (3.7)	1 (0.9)	1.84	3 (2.3)	1 (0.8)	1.02	0.43

*SD, standard deviation; QL, lower quartile; QU, upper quartile; VCA, Violent Cases; NCA, Non-violent Cases; BIS, Barratt Impulsiveness Scale; DSSI, Duke Social Support Index; GDS, Geriatric Depression Scale; BHS, Beck Hopelessness Scale; ULS, University of California Los Angeles Loneliness Scale; DSM-IV, Diagnostic and Statistical Manual of Mental Disorders, Fourth Edition*.

* *A significant difference between groups, P < 0.05*;

** *P < 0.01*;

*** *P < 0.001*.

In addition, unstable marital status was associated with suicide via a non-violent method (*P* < 0.001) but not with a violent method (*P* = 0.052).

### Psychological, Social Environment, and Life Event Characteristics

As shown in Table 1, there was no difference in the presence of a psychiatric diagnosis among suicide cases (*P* = 0.124). The most common mental disorder in both violent and non-violent suicide cases was mood disorder (36.7 vs. 46.6%).

The presence of a psychiatric diagnosis was overrepresented in the suicide group compared to the control group, and the proportion reached 45.0% of the suicide cases in the violent group (*P* < 0.001) and 54.9% of the suicide cases in the non-violent group (*P* < 0.001). The non-violent suicide cases were more likely to have alcohol use disorder than the living controls (*P* = 0.006), but the difference was not significant in the violent group (*P* =0.175).

Suicide cases were more likely to have a higher degree of impulsivity, loneliness, hopelessness, depression, and lack of social support than living controls in both the violent and non-violent groups (all *P* values < 0.001). However, there were no significant differences in the ULS, DSSI, BHS, BIS, and GDS scores between violent and non-violent suicide cases (all *P* values > 0.05).

Suicide cases in the violent group were associated with a higher quantity of life events than those in the non-violent group (*P* = 0.018). In addition, suicide cases were more likely to have a larger impact on life events than living controls in both the violent and non-violent groups (all *P* values < 0.001).

Those who died by suicide via non-violent methods were more likely to store pesticides at home than those who died by suicide via violent methods (38.5 vs. 63.9%; *P* < 0.001). The violent suicide cases were less likely to store pesticides at home compared to the living controls (*P* = 0.041), but the difference was not statistically significant in the non-violent group (*P* = 0.210).

A higher proportion of the non-violent suicide cases had a family suicide history compared to the living controls (24.8% vs. 11.3%; *P* = 0.004), but the difference was not statistically significant in the violent group (*P* = 0.263). No significant difference was found in family suicide history between violent and non-violent suicide cases.

### Multivariate Conditional Logistic Regression Analysis

Table 2 shows the results of the multivariate analysis. The variable that remained in the model as a risk factor for both violent and non-violent suicide was the severity of depressive symptoms. For non-violent suicide, another risk factor was the degree of hopelessness.

**Table 2:** Conditional logistic regressions of risk factors on violent and non-violent suicide methods.

**Variables**	**VCA vs. VCO**		**NCA vs. NCO**	
	***p***	**OR (95% CI)**	***p***	**OR (95% CI)**
BHS	0.118	1.08 (0.98–1.18)	<0.001	1.14 (1.06–1.22)
GDS	<0.001	1.10 (1.07–1.13)	<0.001	1.06 (1.03–1.10)

*VCA, Violent Cases; NCA, Non-violent Cases; VCO, Violent Controls; NCO, Non-violent Controls; BHS, Beck Hopelessness Scale; GDS, Geriatric Depression Scale; OR, odds ratio; CI, confidence interval*.

*The backward stepwise method was used in this logistic regression model*.

### Comparison of the Suicide Characteristics of Violent and Non-violent Suicide Cases

As shown in Table 3, there was a significant difference in the suicide season between the violent and non-violent suicide cases. Older individuals were more likely to choose violent suicide during spring. Furthermore, most of the 242 suicide cases occurred between 6:00 a.m. and 9:59 p.m. and at home. However, no significant differences were found in suicide time, suicide location, number of previous suicide attempts, or the SIS total score between the violent and non-violent suicide cases.

**Table 3:** Comparison of suicide behaviors of suicide cases via violent and non-violent suicide methods.

**Predictor**	**Violent (*n* = 109)**	**Non-violent (*n* = 133)**	**χ2/*t/z***	***p***
Previous suicide attempts, *n* (%)	24 (22.0)	21 (15.8)	1.54	0.215
SIS total score, mean (SD)	6.74 (2.98)	7.11 (2.68)	1.01	0.315
Suicide at home, *n* (%)	92 (84.4)	120 (90.2)	1.87	0.172
Suicide time, *n* (%)			0.40	0.821
10:00 p.m.-5:59 a.m.	17 (15.6)	24 (18.0)		
6:00 a.m.-1:59 p.m.	68 (62.4)	78 (58.6)		
2:00 p.m.-9:59 p.m.	24 (22.0)	31 (23.3)		
Suicide season, *n* (%)	70 (64.2)	64 (48.1)	17.61	0.001
Spring	50 (45.9)	29 (21.8)		
Autumn	23 (21.1)	31 (23.3)		
Summer	16 (14.7)	38 (28.6)		
Winter	20 (18.3)	35 (26.3)		

*SD, standard deviation; SIS, Suicide Intent Scale*.

### Differences Between Suicide Cases in the Use of Violent and Non-violent Suicide Methods: A Logistic Regression

A binary logistic regression model was constructed to examine the differences between suicide cases in the use of violent and non-violent suicide methods. The independent variables included in the model were age (entered as a continuous variable), pesticide stored at home, suicide season and quantity of stimulating life events. After adjusting for age, the results showed that pesticides stored at home were negatively related to the use of violent methods, suggesting that people were more likely to die by suicide using violent methods if no pesticides were stored at home. Suicides via violent methods occurred more frequently in the spring and were associated with a higher quantity of life events than suicides via non-violent methods (see Table 4).

**Table 4:** Factors related to violent suicide methods: a logistic regression model.

**Variables**	***p***	**OR**	**95% CI**
Age	0.196	1.02	0.99–1.06
Suicide season	0.001		
Spring		1	ref
Autumn	0.016	0.40	0.19–0.85
Summer	<0.001	0.24	0.11–0.53
Winter	0.002	0.31	0.15–0.66
Pesticides stored at home			
Yes	<0.001	0.34	0.19–0.60
No		1	Ref
Life events stimulating quantity	0.019	1.01	1.00–1.02
Constant	0.411	0.31	

*OR, odds ratio; CI, confidence interval*.

*The Enter method was used in this logistic regression model*.

## Discussion

This study revealed the following major findings: (1) among elderly adults in rural China, the number of suicides via non-violent methods was higher than that via violent methods; (2) as a non-violent suicide method, pesticide ingestion was the leading method of suicide used by elderly individuals in rural China, and the most common violent suicide method was hanging; (3) among elderly individuals, those who died by suicide by means of violent methods tended to be older, be more likely to die by suicide during the spring, have a higher quantity of stimulating life events, and have no pesticides stored at home than those who died by suicide by means of non-violent methods; and (4) the major risk factor for both violent and non-violent suicide was the severity of depressive symptoms. For non-violent suicide, the degree of hopelessness was another major risk factor.

Certain demographic factors were related to suicide via violent methods. Previous studies have indicated that those who attempted violent suicide were more likely to be older than those who attempted non-violent suicide (31), and elderly individuals who died by suicide were more likely to live without a spouse than younger individuals who died by suicide (32). In this study, we found that elderly individuals with unstable marital status were more likely to suicide via a non-violent method than via a violent method. Suicide prevention programs are needed to develop strategies specialized by suicide method to identify high-risk individuals in rural China.

Availability and acceptability were key factors influencing the choice of suicide method among adults aged 65 years and older (33). Pesticide ingestion is the most common suicide method, and it has a long history in rural China (10). In this study, pesticide ingestion (51.7%) was still the most favored suicide method among older adults in rural China. According to the logistic regression analyses of differences between suicide cases in the use of violent and non-violent suicide methods, individuals who had pesticides stored at home were less likely to die by suicide using violent methods. Although pesticide ingestion is a non-violent suicide method, it is still highly lethal. Pesticides are easily accessible in China, which is the largest producer and consumer of pesticides worldwide (34). Strong evidence suggests that limiting general access to highly hazardous pesticides (so-called means restriction) is an effective approach for suicide prevention (35). For example, in Sri Lanka, replacing highly toxic pesticides with less hazardous pesticides and integrated pest management has resulted in a 75% reduction in total suicides (36). In addition, restricting the availability of common and highly lethal suicide methods can reduce both method-specific and all-cause suicide rates (37).

Previous research found that seasonality was related to suicide via both violent and non-violent methods (38). Suicide exhibits pronounced seasonality in rural areas (39). In our study, older individuals who died by suicide were more likely to choose violent methods during spring than during other seasons. Individuals who died by suicide using pesticide ingestion accounted for 94.0% of all non-violent suicides in this study. Given that the use of pesticides increases according to agricultural work, which exhibits seasonality, the availability of pesticides may also display certain seasonality. In addition, previous studies have found that the duration of daily sunshine is significantly associated with seasonal variations in violent suicides and has a direct effect on violent suicides (40). During the spring, the sunshine hours per day are shorter in China. Sunshine may interact with brain serotonin systems and impact serotonin-related behaviors, such as depression, impulsiveness, and aggression, which are known to play key roles in suicidal behavior (41, 42). Therefore, different suicide prevention strategies should be designed according to different seasonal patterns in rural China.

Other suicide behavior features include suicide timing and location. In this study, we found that 60.3% of the suicides occurred between 6:00 a.m. and 1:59 p.m., and 87.6% of the suicides occurred at home, but there were no significant factors identified for violent suicide methods; this result highly differed from former research investigating younger individuals who died by suicide in rural China that showed that most suicides occurred at nighttime and that suicides via violent methods were more likely to occur outside than inside (43).

Previous studies have shown that individuals who died by suicide in later life tended to exhibit high suicide intent (6, 44). A previous suicide attempt is an important indicator of suicide (13, 44). Furthermore, previous research found that committing violence was associated with a high suicide intent in individuals with previous suicide attempts (45). In the present study, we did not find statistically significant differences in suicide intent and a prior history of suicide attempts between the violent and non-violent groups among elderly individuals, which is inconsistent with a study that reported that a relatively high suicide intent score was associated with the use of violent methods in rural China (16). Our data indicated that suicide intent and previous suicide attempts might not determine the use of violent or non-violent suicide methods among elderly individuals in rural China.

The influence of life events on suicide was measured by the quantity of stimulating life events in this study. We found most of the elderly individuals who died by suicide (93.3%) had experienced multiple life events during the year before death. According to the logistic regression analyses of differences between suicide cases in the use of violent and non-violent suicide methods, quantity of life events was positively associated with violent suicide methods. Thus, suicide cases with a high quantity of life events were more likely to choose violent suicide methods than those with a low quantity. In this case, intervention work should be targeted to prevent violent suicides with high lethality and offer financial and medical help to rural residents who are experiencing despair or overwhelming life changes.

In the present study, the severity of depressive symptoms provided a more powerful predictor of suicide. Previous studies found that mental disorders were also a risk factor for suicide in elderly individuals and were associated with violent suicide methods (43, 45). Our results showed that the elderly suicide cases were more likely to have mental illness compared to the living controls in both the violent and non-violent groups. However, the presence of categorical diagnoses did not remain in the final regression model either for violent or for non-violent suicide methods. In addition, there were no significant differences in the distribution of mental disorders and GDS scores between the violent and non-violent suicide cases; this result differs from that found in younger individuals with mental disorders who tend to use violent suicide methods (43).

Previous studies indicated that substance abuse was associated with suicide (46), but our study did not find that. In this study, we observed that the elderly suicide cases were more likely to have alcohol use disorder compared to the living controls in the non-violent groups. However, alcohol use disorder did not remain in the final regression model for non-violent suicide methods. In addition, there were no significant differences in the distribution of alcohol use disorder between the violent and non-violent suicide cases. Our finding is inconsistent with a study in US that in older adulthood, individuals were more likely to drink alcohol when they used poisoning (compared with firearm or hanging) (47). A previous study also indicated that acute use of alcohol may influence the selection of a suicide method based on its lethality (48).

Previous studies (4, 16, 32, 49) found that loneliness, hopelessness, impulsiveness and low social support were risk factors for suicide in elderly individuals. In the present study, suicide cases were more likely to have a higher degree of impulsivity, loneliness, hopelessness, and lack of social support than living controls in both violent and non-violent groups. However, only hopelessness remained in the final regression model for non-violent suicide methods, not for violent suicide methods. This result is inconsistent with a study that 15–34 age suicides with a high degree of hopelessness were more likely to suicide with violent methods in rural China. A previous study in rural Shandong also indicated that degree of hopelessness was the most common risk factor for violent and non-violent suicides. These differences may be due to age; therefore, different suicide prevention strategies should be adopted for different age groups.

## Conclusion

Our findings may have important implications for suicide prevention in elderly individuals in rural areas in China. However, this study focused specifically on violent and non-violent methods among elderly individuals in rural China and found some specific characteristics in older individuals who died by suicide that differed from those in younger individuals who died by suicide in rural China. More importantly, depression was the major risk factor for suicide in both violent and non-violent elderly suicides. Suicide prevention measures that focus on depression among this vulnerable population are urgently needed. Furthermore, we found that pesticide ingestion, which is a non-violent suicide method, remained the most frequently used suicide means among elderly individuals in rural China, and factors related to violent suicide methods were a lack of pesticides stored at home, spring season, and a high quantity of stimulating life events. Clearly, further efforts should be made to inform suicide prevention strategies according to the risk factors for the suicide methods and the characteristics of the elderly individuals who have died by suicide in rural China.

## Limitations

This study has several limitations. The key limitation was that the psychological autopsy method has methodological shortcomings. It is not noted that the tools used to obtain information from informants were not validated in informants but rather in individuals (who, in this study, died by suicide). Informant answers may be influenced by their personal mental state, relationship to the deceased, etc. Information errors and bias due to informants' subjective observations and recall mistakes might exist.

In this study, we did not focus on the role of media, which is sometimes an important factor impacting suicidal behavior and methods (50). It was difficult to collect information on whether elderly suicide cases watched programs, read the newspaper or used the internet related to suicide due to the inherent methodological limitation of using proxy-based information.

To minimize this limitation, much of the information in this study was based either on prior documented data or *post hoc* independent psychiatric evaluation. The validity of this procedure with regard to the type of variables used in the current study has been well-demonstrated in previous study (51).

## Data Availability Statement

The datasets presented in this study can be found in online repositories. The names of the repository/repositories and accession number(s) can be found in the article/supplementary material.

## Ethics Statement

This study was approved by the Institutional Review Boards of Shandong University, Central South University, and Guangxi Medical University. Written informed consent was obtained from all who provided information (including the next-of-kin who lived with the suicide victim, and a friend, a neighbor, or a remote relative) about the individuals who died by suicide.

## Author Contributions

LZ, Z-YM, and C-XJ did the study design, coordinated the study, and recruited participants. R-TZ analyzed the study data and drafted the manuscript. LZ edited the manuscript. All authors contributed to the article and approved the submitted version.

## Conflict of Interest

The authors declare that the research was conducted in the absence of any commercial or financial relationships that could be construed as a potential conflict of interest.
